# Managing children with brain tumors during the COVID-19 era: Don’t stop the care!

**DOI:** 10.1016/j.csbj.2021.01.005

**Published:** 2021-01-12

**Authors:** Michele Antonio Capozza, Silvia Triarico, Giorgio Attinà, Alberto Romano, Stefano Mastrangelo, Palma Maurizi, Paolo Frassanito, Federico Bianchi, Tommaso Verdolotti, Marco Gessi, Mario Balducci, Luca Massimi, Gianpiero Tamburrini, Antonio Ruggiero, On behalf of the Gemelli Pediatric Neuro-Oncology Tumor Board

**Affiliations:** a Pediatric Oncology Unit, Fondazione Policlinico Universitario A. Gemelli IRCCS, Università Cattolica Sacro Cuore, Rome, Italy; b Pediatric Neurosurgery Unit, Fondazione Policlinico Universitario Agostino Gemelli IRCSS, Università Cattolica Sacro Cuore, Rome, Italy; d UOC Radiologia e Neuroradiologia, Fondazione Policlinico Universitario “A. Gemelli” IRCCS, Rome, Italy; e Neuropathology Unit, Department of Pathology Fondazione Policlinico Universitario “A. Gemelli” IRCCS, Università Cattolica Sacro Cuore, Rome, Italy; c UOC Radioterapia Oncologica, Fondazione Policlinico Universitario “A. Gemelli” IRCCS, Università Cattolica Sacro Cuore, Rome, Italy

**Keywords:** COVID-19, Brain tumors, Children, Care

## Abstract

The COVID-19 pandemic has substantially stressed health care systems globally, subsequently reducing cancer care services and delaying treatments. Pediatric populations infected by COVID-19 have shown mild clinical symptoms compared to adults, perhaps due to decreased susceptibility. Several scientific societies and governments have released information on the management of patients with cancer, wherein they warn against exposure to SARS-CoV-2 infection and suggest continuing treatment. To determine the best diagnostic and therapeutic approach, multidisciplinary tumor boards should convene regularly, including through conference calls and telematics platforms. A prompt diagnostic workup may reduce children's suffering and prevent loss of confidence in the health care system among parents. Moreover, ensuring adequate support and information regarding measures for preventing SARS-CoV-2 infection in pediatric patients and their families is essential for avoiding panic and excessive stress, allowing early reporting of any suspected symptoms of cancer and, in turn, facilitating early diagnosis and prompt modulation of treatment.

## 1 Introduction

In December 2019, the Coronavirus Disease (COVID-19) caused by a novel zoonotic coronavirus (SARS-CoV-2) emerged from Wuhan, China and rapidly escalated into a global pandemic. Presently, Europe and the USA have become the epicenter of the pandemic, with many countries (e.g., Italy, France, Spain, and the United Kingdom) having imposed lockdowns to limit social contact and contain the outbreak [1], [2].

The COVID-19 pandemic has affected the ability of health care systems worldwide to properly address the new health emergency. To increase the resources, such as hospitals beds and medical staff, procedures deemed elective or non-urgent have been delayed, postponed, or canceled. Based on data regarding the COVID-19 outbreak first in China and then worldwide, children aged less than 10 years old have been found to exhibit significantly lower susceptibility to SARS-CoV-2 infection compared to adults [3], [4].

Apart from the low incidence of infection and death, pediatric populations have been reported to present with a less severe illness course. Moreover, one study showed that children exhibit milder clinical symptoms and a shorter disease duration compared to the elderly population [5].

A systematic review conducted by Christophers et al. revealed that majority of children with SARS-CoV-2 presented with either no symptoms or a single symptom, with 75% of asymptomatic children exhibiting abnormal chest radiography or computed tomography findings [6].

In addition, recent reports have focused on pediatric patients presented with an hyperinflammatory syndrome with features of Kawasaki disease termed multisystem inflammatory syndrome in children (MIS-C). This clinically severe illness characterized by fever, increase of inflammatory markers, and multiorgan dysfunction in the setting of recently proven or probable SARS-CoV-2 infection can have overlapping features with Kawasaki disease although it should be considered as a distinct entity [7].

Presently, the extent to which emergency measures need to be maintained has been unclear, while many concerns have been raised regarding the delivery of care for children with chronic diseases and cancer. Published evidence has shown that children with cancer have comparable risk for SARS-CoV-2 infection as healthy children [8].

Moreover, children and young people who had undergone prolonged periods of immunosuppressive chemotherapy do not have increased risk for severe COVID-19 infection [9].

Despite the absence of clear guidelines regarding the delivery of care for children with cancer, pediatric anticancer treatments should not be interrupted or delayed. Conversely, active strategies to avoid situations that increase the risk for infection and hygienic educational programs must be guaranteed and implemented for immunosuppressed children with cancer [10]. During the current COVID-19 pandemic, many hospitals are responding by reducing cancer care services and delaying treatments. However, priority should be given to children in whom cancer treatment is likely to be curative.

## 2 Potential mechanisms of children’s low susceptibility to SARS-CoV-2

Although several hypotheses have attempted to explain why children have low susceptibility to SARS-CoV-2, the real reasons for the lower frequency and mild symptoms in children have yet to be established [11].

Children’s vaccines are supposed to induce cross-reactivity against SARS-CoV-2 [12]. Unfortunately, antisera and T cells from mice immunized with different vaccines did not develop cross-reactivity against SARS-CoV-2. Therefore, other factors, including the inability of children to mount a prompt hormonal response, upregulated anti-inflammatory cytokines in the lungs, or a weaker immune reactivity to viral infections, may explain the phenomenon of reduced susceptibility among children affected by SARS-CoV-2 [13].

Recently, the distribution and functioning of viral receptors has been suggested as key factors for age-related differences in the incidence and severity of the SARS-CoV-2 infection. Previous studies have demonstrated that some coronaviruses, such as SARS-CoV-2, bind to angiotensin-converting enzyme-2 (ACE2) cell receptors in humans. However, ACE2 expression in the rat lung reduces dramatically with age, a finding seemingly in contrast with the low susceptibility of children to COVID-19 [3], [14].

Research on the SARS-CoV-2 entry and infectivity has identified Neuropilins as entry factors due to their high expression on epithelial cells that line the respiratory tract and their function in enabling cell, vascular, and tissue penetration. In particular, the activation of the Neuropilin-1 (NRP1), a transmembrane receptor which acts primarily as a co-receptor for different ligands and highly expressed in the respiratory and olfactory epithelium, can induce multiple effects, including cell proliferation and angiogenesis. Therefore, NRP1 could act as a potential host factor for SARS-CoV-2 infection by promoting the interaction between the viral spike (S) protein and the surface of human cells [15].

Other theories include evidence that children have fewer underlying medical disorders, healthier respiratory tract mucosae, and lower reactivity of the innate immune response compared to adults in whom the prompt and vigorous immune response activates a detrimental cascade immune-associated with acute respiratory distress [16].

Another proposed mechanism is related to the role of the thymus and its natural progressive decline over time. Thymic involution generally starts after the first year of age, with an accelerated reduction soon after puberty. Notably, progressive thymic decline produces a significant reduction in T cell output, with those over 50 years old possible having a T cell supply sustained only by existing naïve and memory T cells. Moreover, a comparison of functional CD4 and CD8 T cell changes with age showed that CD8 T cells are more susceptible to the effects of aging, with a more sensible reduction in their functional memory subset. CD8 T cells function primarily in antiviral immunity give their ability to recognize and destroy virus-infected cells. Overall, the aforementioned data appear to suggest that thymus involution might be involved in the weaker immune responses generally observed among elderly individuals [13], [17].

## 3 Childhood tumors and SARS-CoV-2

The risk for severe SARS-CoV-2 disease increases with advanced age, smoking habits, and comorbidities, such as cancer [18]. In fact, Liang et al., who analyzed the clinical characteristics of 18 adults in a cohort of 1590 Chinese cases, observed that patients with cancer had a higher risk for severe events (e.g., intensive care unit admission or death) than those without cancer due to their immunosuppressed status [19].

The management of patients with cancer during the COVID-19 epidemic must consider their increased risk for severe disease, subsequently providing appropriate education regarding hand hygiene, infection control measures, high-risk exposure, signs and symptoms of COVID-19, minimizing outpatient visits, and the use of telemedicine [20], [21].

Although children with cancer are considered a high-risk population, only a few published studies have described outcomes of SARS-CoV-2 infection in children with cancer. In Lombardia (Italy), pediatric onco-hematologic centers registered 21 cases of SARS-CoV-2 infection between February and April 2020, among whom only nine with a positive swab had typical symptoms, such as cough, fever, and/or colds. Moreover, 10 of them were affected by leukemia, 2 by lymphoma, 9 by solid tumors, including a case with brain cancer. Chemotherapy was provided to 15 patients, while 10 cases required delay of the therapy, reduction of administered drug doses, or delayed surgery. The patient with Hodgkin's lymphoma, previously treated with radiotherapy, developed mild pneumonia [22]. Interestingly, a decrease in new diagnoses (reduced by approximately 55% compared to the previous year) was observed, perhaps due to delayed access to healthcare facilities, a change in parental behavior toward avoiding SARS-CoV-2 infection, and a reduction in patient migration between regions or states [23].

In their study on pediatric cancer patients (aged 0–18 years) in Madrid, De Rojas et al. identified 15 patients with SARS-CoV-2 infection: 73% with hematological neoplasms and 27% with solid tumors. Among such patients, 60% received chemotherapy within 15 days prior to the diagnosis of SARS-CoV-2 infection, while 40% required a modification or delay in their chemotherapy. Most patients received hydroxychloroquine and azithromycin, tocilizumab, lopinavir–ritonavir, corticosteroids, and remdesivir in different combinations as therapy, with no adverse effects related to the COVID 19 treatment having been registered. Two patients needed oxygen support, while one required invasive ventilation support [24].

A screening plan was performed at the Memorial Sloan Kettering Cancer Center to evaluate the risk associated with SARS-CoV-2 infection. Accordingly, such data revealed that pediatric patients with cancer had low overall morbidity due to SARS-CoV-2, with only 5% of infected patients requiring hospitalization for severe symptoms [25].

On the other hand, reports from pediatric oncology centers in France have registered worse outcomes. Notably, 5 out of 33 patients with cancer infected with SARS-CoV-2 required intensive care. Among the five patients, three were immunosuppressed after recently receiving chemotherapy for relapsed B-cell lymphoblastic leukemia, two received allogenic stem cell transplantation, and finally one had a high-grade glioma with poor clinical conditions [26].

Therefore, the general management of children with cancer is based on prudential measures for avoiding exposure to SARS-CoV-2 infection while trying to guarantee treatment. Common habits generally adopted by children with cancer continue to be valid, such as frequent hand washing, use of face masks, and avoiding crowded places or contact with individuals having respiratory disorders. Moreover, new rules should be considered for all children with cancer, such as providing only one parent access to pediatric units, physically isolating children in a single bedroom, adopting online medical counseling for limiting hospital accesses, and an appropriate identification and treatment of severe cases [27], [28].

## 4 Impact of COVID-19 pandemic on pediatric cancer clinical trials

Chemotherapy has made a significant contribution to the successful treatment of childhood cancer and has led to a marked increase in the cure rate of most of these diseases. Clinical research has been fundamental to enhancing medical treatment progress and patient care but the current COVID-19 pandemic has led to the shift of priority to SARS-CoV-2 affected patients.

This has had a severe impact on the development of clinical research in other fields, such as in cancer clinical trials. Furthermore, pharmaceutical companies have devoted their economic resources to the development of technologies and drugs against COVID-19 reducing resources for clinical research in the field of oncology.

In order to limit the negative impact on patients, the EMA (European Medicines Agency) and many scientific societies have issued recommendations and guidelines in order to support the conduct of clinical trials in this patient setting while continuing to ensure patient safety. Nevertheless, a negative impact has been reported on ongoing and starting studies, especially in recruitment and enrollment, with protocol deviations as patients miss hospital visits as well as delayed data reporting or monitoring activity [29], [30].

However, health systems and healthcare staff have been able to respond to the evolving COVID-19 pandemic. At present, the results across all clinical trial activities are better than what was reported in early phases of the outbreak (April 2020) with no moderate or severe impact on singular activities. The impact of COVID-19 on ongoing trials is especially related to the sites’ ability to recruit and enroll patients and the increasing costs of personal protective equipment [31].

At this time of health emergency we must not forget that cancer is still the main disease-related cause of death in children. Therefore, research in this field needs to continue to be supported both scientifically and economically [32]. The optimal use of modern technologies such as video, apps or mobile phones, electronic consent and telemedicine consultation could replace some protocol mandatory hospital visits and provide an efficient tool to greatly reduce the bureaucratic burden and methods of data entry [33].

## 5 Effects of COVID-19 on pediatric patients with brain tumors

The activities of each cancer discipline has been adversely affected by the COVID-19 pandemic. In an effort to prepare for future outbreaks of the COVID-19 pandemic, health care systems are establishing plans, including limitations to the operating rooms, reduction of elective or non-urgent procedures, and delay of medical treatments. At present, no clear indications have shown that active treatment of children with cancer should be interrupted. However, postponing considerably intensive treatments, such as therapies involving bone marrow transplantation, may be reasonable and should be approached on a case-by-case basis according to each clinical and epidemiological condition [10].

Moreover, malignant pediatric brain tumors are characterized by rapid growth requiring prompt diagnosis and appropriate treatment. Therefore, a delay or modification in therapy may compromise their effectiveness and lower patients' survival rates. Flores et al. reported that delayed diagnosis of brain tumors are correlated with a worse prognosis. A prolonged pre-diagnostic symptomatic interval has negative and severe consequences, such as death or serious brain damage following the increase in intracranial pressure [11]. Children with delayed brain cancer diagnosis have a higher risk for developing irreversible neurological disabilities from acquired brain lesions, such as loss of vision and endocrinopathies [28], [34], [35], [36]. Thus, early diagnosis remains a priority for minimizing neurological morbidity, preventing the onset of acute symptoms and sequelae, allowing extensive tumor resection, and reducing parents’ psychological discomfort and health costs. As such, for cases with a suspected brain tumor, promptly contacting the pediatric cancer center in order to optimize further procedures and avoid delays of treatment should be mandatory. A prompt diagnostic workup reduces children's suffering and prevents parents’ loss of confidence in the health care system [16].

However, there is no doubt that the COVID-19 pandemic is still causing delayed and suboptimal treatment for patients with cancer. This could lead to excess deaths from cancer in the next year or two both in adults and children [37].

Changes in the decision-making process for children with brain tumors due to fear and pressure of SARS-CoV-2 infection could negatively influence their final outcome. Never have multidisciplinary tumor boards serve as crucial a role during the current COVID-19 outbreak. All pediatric cases diagnosed with a new brain tumor should be discussed in a multidisciplinary tumor board in order to determine the optimal therapy for each child. Therefore, medical counseling and multidisciplinary tumor boards need to continue to be active during the ongoing current COVID-19 pandemic by adopting new technology solutions, such as call-conferences and telematic platforms, to ensure that patients receive the best diagnostic and therapeutic approach that would improve their survival chances [17].

Surgery still remains the first treatment approach given its curative potential for non-malignant tumors and ability to obtain tumor tissue for the pathological diagnosis of malignant tumors. Therefore, health care systems should ensure dedicated cancer operating lists to mitigate the pressures of the COVID-19 pandemic. Adjuvant radiotherapy and/or chemotherapy can then follow on a case-by-case basis.

## 6 Conclusions

In the absence of underlying medical disorders, children infected by SARS-CoV-2 develop milder and shorter clinical symptoms than adults without requiring intubation for severe acute respiratory distress and intensive care unit admission. Available data show that children are less susceptible to SARS-CoV-2. Although the reasons for such low susceptibility remain unclear, several theories have been formulated. Moreover, little is known regarding the impact of SARS-CoV-2 infection on children with cancer, with the management of this vulnerable subset of patients being based on local and unstructured measures. Several scientific societies and governments have provided information on the management of patients with cancer; nonetheless, clear warnings are essential to avoid inappropriate behavior and initiatives. Educational hygiene programs remain fundamental and must be implemented for the management of the current and the future infectious epidemics. Published studies suggest that cancer treatments for pediatric patients should continue, without substantial interruptions or modifications. Multidisciplinary tumor boards have to convene regularly, including through call-conferences and telematics platforms. Given the current circumstances surrounding the pandemic, ensuring adequate support and information regarding measures for preventing SARS-CoV-2 infection in pediatric patients and their families is essential. Adequate knowledge and information are essential to avoid panic and excessive stress, thereby allowing early reporting of any suspected symptoms of cancer and, in turn, facilitating early diagnosis and prompt modulation of treatment.

## Funding

This research received no external funding.

## CRediT authorship contribution statement

**Michele Antonio Capozza:** Methodology, Validation, Writing - original draft. **Silvia Triarico:** Methodology, Validation, Data curation, Writing - original draft. **Giorgio Attinà:** Conceptualization, Writing - original draft. **Alberto Romano:** Validation, Data curation, Writing - review & editing. **Stefano Mastrangelo:** Methodology, Data curation. **Palma Maurizi:** Methodology, Data curation. **Paolo Frassanito:** Data curation, Writing - review & editing. **Federico Bianchi:** Methodology, Writing - review & editing. **Tommaso Verdolotti:** Conceptualization, Data curation, Writing - review & editing, Supervision. **Marco Gessi:** Data curation, Writing - review & editing, Supervision. **Mario Balducci:** Conceptualization, Writing - review & editing, Supervision. **Luca Massimi:** Conceptualization, Writing - original draft, Writing - review & editing. **Gianpiero Tamburrini:** Conceptualization, Writing - review & editing, Supervision. **Antonio Ruggiero:** Conceptualization, Methodology, Writing - original draft, Supervision.

## Declaration of Competing Interest

The authors declare that they have no known competing financial interests or personal relationships that could have appeared to influence the work reported in this paper.
